# Statin Intolerance: the Clinician’s Perspective

**DOI:** 10.1007/s11883-015-0552-3

**Published:** 2015-10-21

**Authors:** Tomáš Stulc, Richard Ceška, Antonio M. Gotto

**Affiliations:** 3rd Department of Internal Medicine, 1st Faculty of Medicine, Charles University, U Nemocnice 1, CZ 128 21 Praha 2 / Prague, Czech Republic; Weill Cornell Medical College, New York, 1305 York Avenue, Y-807, New York, NY 10021 USA

**Keywords:** Statin, Statin intolerance, Muscle side effects, Myalgia, Low-dose statin therapy

## Abstract

Muscle problems and other adverse symptoms associated with statin use are frequent reasons for non-adherence and discontinuation of statin therapy, which results in inadequate control of hyperlipidemia and increased cardiovascular risk. However, most patients who experience adverse symptoms during statin use are able to tolerate at least some degree of statin therapy. Given the profound cardiovascular benefits derived from statins, an adequate practical approach to statin intolerance is, therefore, of great clinical importance. Statin intolerance can be defined as the occurrence of myalgia or other adverse symptoms that are attributed to statin therapy and that lead to its discontinuation. In reality, these symptoms are actually unrelated to statin use in many patients, especially in those with atypical presentations following long periods of treatment. Thus, the first step in approaching patients with adverse symptoms during the course of statin therapy is identification of those patients for whom true statin intolerance is unlikely, since most of these patients would probably be capable of tolerating adequate statin therapy. In patients with statin intolerance, an altered dosing regimen of very low doses of statins should be attempted and, if tolerated, should gradually be increased to achieve the highest tolerable doses. In addition, other lipid-lowering drugs may be needed, either in combination with statins, or alone, if statins are not tolerated at all. Stringent control of other risk factors can aid in reducing cardiovascular risk if attaining lipid treatment goals proves difficult.

## Introduction

Statins effectively decrease cardiovascular risk, and cholesterol lowering with statins has become a cornerstone of cardiovascular disease prevention for a wide range of patients [1]. Despite this, adequate use of statins is limited by adverse symptoms in many patients [2–4], which leads to statin discontinuation in some patients, and low adherence to therapy in others. The issue of statin intolerance is, therefore, of great clinical importance.

Despite the existence of statin intolerance having been widely acknowledged, a large degree of variability remains as to what is considered to be statin intolerance. In addition, there is significant uncertainty regarding the actual incidence, and there is insufficient knowledge concerning the best therapeutic approaches to the problem. Most cases of statin intolerance are related to muscle complaints [5–7], with increased liver or muscle enzymes [8], various neurological symptoms [9] and other problems being much less frequent. The glycemic effects of statins are occasionally included as a symptom of statin intolerance; although, they are undesired side effects rather than serious findings necessitating discontinuation of therapy in individual cases. Moreover, as we have previously discussed elsewhere [10], the diabetogenic effects of statins are generally overestimated.

It should be noted that all of the mentioned symptoms can stem from a number of different causes and are often unrelated to actual statin use. However, even among patients with true statin-related symptoms, many can tolerate lower doses of the same statin, or perhaps a different statin. Establishing a diagnosis of statin intolerance is therefore less straightforward than it might appear, and an adequate therapeutic approach is more complex than simple discontinuation of statin therapy.

As a result of these complexities, statin intolerance is currently gaining increased attention and several guidelines [5, 11, 12] and review papers [13–16] have recently been published, thereby providing an in-depth discussion of this complicated, and often controversial, topic. Practicing physicians, however, may find the aforementioned recommendations somewhat lengthy and difficult to implement in their daily practice. In this review, we propose a practical definition of statin intolerance and outline a therapeutic approach to patients with this condition.

### Definition of Statin Intolerance

In general terms, statin intolerance can be defined as the occurrence of (1) adverse symptoms perceived by the patient to be unacceptable, and/or (2) laboratory abnormalities suggesting undue risk, which are attributed to statin therapy and lead to its discontinuation. For practical purposes, descriptions regarding acceptability of symptoms, attributability to statin therapy, and the degree of intolerance, need to be better defined.

Most cases of statin intolerance are related to patient complaints; the discontinuation of therapy due to laboratory abnormalities is far less common. Thus, in most cases, decisions pertaining to statin intolerance are patient decisions. In this context, it is important to note that some degree of adverse symptoms might be tolerated by patients and it does not necessarily imply intolerance of therapy. Statin intolerance is not simply the occurrence of symptoms in general, but rather those symptoms that are perceived to be unacceptable. Hence, the patient’s subjective assessment of perceived risks and inconveniences, versus the benefits of therapy, are at the core of an effective approach to the issue of statin intolerance.

When experienced during the course of statin therapy, myalgia and other adverse symptoms are often unrelated to treatment, and most patients with a history involving episodes of these symptoms are able to tolerate adequate statin therapy [17, 18]. *Identifying true cases of statin intolerance is*, *therefore*, *of great practical importance in order to avoid unnecessary discontinuation of statins from patients who would otherwise benefit from them*. However, evaluating the likelihood that the adverse symptoms are causally related to statins is often a difficult task (Table 1). Close temporal association to statin therapy is an important feature that suggests causality. Symptoms are more likely to be attributable to statins if they appear within the first month of statin therapy, improve upon discontinuation, and reoccur after readministration [3, 4]. Consequently, dechallenge–rechallenge testing is an important evaluation tool when assessing statin intolerance. Similarly, regional distribution and the type of pain/complaints are important for assessing their association with statin therapy. A causal relation to statins is more likely in cases of symmetrical involvement of large muscle groups (especially proximal lower limbs) or in cases with widespread involvement; a causal relationship is less likely when symptoms are asymmetrical or involve small isolated regions. Typical complaints involve muscle pain, tenderness or cramps, and weakness during exertion, while joint or tendon pain, muscle tingling and twitching, or shooting pain, suggest causes other than statin therapy. In addition, factors known to be associated with increased risk of statin intolerance, as well as contributing factors that may precipitate symptom manifestation, such as hypothyroidism [19], drug interactions, and vitamin D deficiency [20], should be carefully evaluated (Table 2). It is also important to rule out conditions that are associated with symptoms that mimic statin intolerance (musculoskeletal disorders, in particular). The American College of Cardiology has recently developed an ACC Statin Intolerance App to aid clinicians in evaluating and managing patients who report muscle symptoms while on a statin (available at http://www.acc.org/StatinIntoleranceApp).

**Table 1:** Evaluating the likelihood of an association between muscle complaints and statin therapy

	Causal relation of symptoms to statin therapy	
	Likely	Unlikely
Regional distribution	• symmetrical • widespread or large muscle groups involvement (proximal lower limbs, calves, proximal upper limbs)	• asymmetrical, unilateral • small isolated regions
Characteristics of the complaint	• muscle pain, tenderness, cramps, stiffness • muscle weakness or heaviness during exertion	• shooting pain • muscle tingling or twitching • joint or tendon pain
Temporal association to statin therapy	• symptoms appear within 4 weeks of initiation of statin	• symptoms appear after >12 weeks of initiation of statin
	(*appearance of symptoms within 4*–*12 weeks contributes marginally to the evaluation of causality*)	
Dechallenge/ rechallenge testing	• symptoms improve within 4 weeks upon discontinuation of statin • symptoms reoccur within 4 weeks after readministration of statin	• late or no improvement of symptoms upon discontinuation of statin • late or no reoccurrence after readministration of statin

Adapted from [3, 4, 6]

**Table 2:** Factors associated with increased risk of statin intolerance

• History of muscular symptoms with other lipid-lowering therapies • History of unexplained muscular symptoms • History of unexplained creatine kinase elevation • Family history of muscular symptoms with lipid-lowering therapy • Strenuous exercise • Hypothyroidism • Vitamin D deficiency • Drug interactions (gemfibrozil, macrolides, azole antifungals, verapamil, amiodarone, protease inhibitors, cyclosporine) • Advanced age • Female gender • Low body mass index • Alcohol abuse	

Adapted from [3, 11]

Given the specifications mentioned above, we propose the following *definition of statin intolerance for use in clinical practice*:

Statin intolerance is the occurrence of (1) adverse symptoms perceived by the patient to be unacceptable, and/or (2) laboratory abnormalities suggesting undue risk, which are attributed to statin therapy and lead to its discontinuation.

To be attributed to statins:

- symptoms or abnormalities should be temporally associated with the initiation of statin therapy, improve upon discontinuation, and reoccur after the readministration of therapy, and
- known precipitating factors and conditions with similar presentations should first be excluded. These primarily include musculoskeletal diseases, hypothyroidism, vitamin D deficiency, strenuous exercise, intercurrent illness, or drug interactions (e.g., azole antifungals, macrolide antibiotics, verapamil).

Regarding the degree of statin intolerance, it can be classified as partial or complete (additional information provided below).

Mild symptoms should not be considered intolerance, provided they are deemed acceptable by the patient.

Among patients presenting with statin intolerance, there is great variability regarding the number and doses of statins they are unable to tolerate. Some patients are intolerant of virtually all statins, even in low doses; others only experience adverse symptoms with a particular statin, or only with the highest doses of particular statins. With this in mind, *we propose two degrees of statin intolerance for consideration*:

- *complete statin intolerance*: the inability to tolerate a minimum of three statins at their usual lowest daily starting doses, and
- *partial statin intolerance*: the inability to tolerate statin therapy in the form and dosages required to achieve treatment goals (including the highest doses of potent statins, if needed).

For the purposes of this definition, the lowest daily starting doses of statins are proposed as rosuvastatin 5 mg, atorvastatin 10 mg, simvastatin 20 mg, lovastatin 20 mg, pravastatin 40 mg, fluvastatin 40 mg, and pitavastatin 2 mg.

Partial intolerance is pragmatically defined with respect to the therapeutic needs of individual patients. *Inability to tolerate some statins*, *or some doses*, *should not be considered as statin intolerance*, *provided it does not interfere with the achievement of treatment goals*.

### Therapeutic Approach to Statin Intolerance

**Fig. 1:**
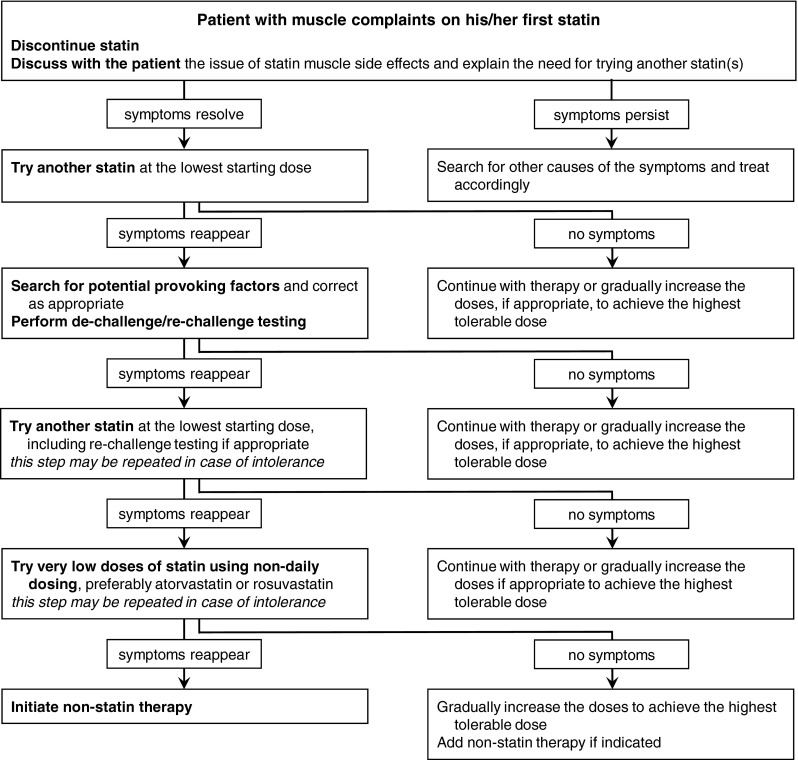
Flowchart for evaluation and management of patients with statin-associated muscle complaints

Most patients who experience adverse symptoms when using statins are able to tolerate at least one statin, albeit sometimes only when administered in an altered dosing regimen. Given the profound cardiovascular benefits of statins, *statin therapy remains the mainstay of lipid*-*lowering treatment for most of these patients* (Fig. 1).

In many patients, adverse symptoms are unrelated to statin usage, especially in those with atypical and intermittent presentations following long periods of treatment. *The first step* in approaching patients who experience adverse symptoms during the course of statin therapy *is therefore to assess whether the symptoms are likely attributable to statins*. This includes obtaining a complete history of symptoms, evaluation of risk factors for statin intolerance (Table 2), temporary withdrawal of statins followed by a rechallenge, as well as seeking other causes of the symptoms [5]. If statin intolerance appears unlikely, the patient can probably tolerate adequate therapy with the same, or alternative, statin.

Likewise, *potential provoking factors* such as hypothyroidism, vitamin D deficiency, or drug interactions *should also be evaluated*, as correcting these problems may improve statin therapy tolerance.

*In patients with statin intolerance*, *very low doses of statins administered via an altered dosing regimen should be attempted* and, if tolerated, should be gradually increased to achieve the highest tolerable doses. With this cautious approach, the majority of patients are able to tolerate at least some degree of statin therapy. In addition, *other lipid*-*lowering drugs may be needed* to achieve the appropriate targets. The principles of lipid-lowering therapy in cases of statin intolerance are discussed in the following sections.

Lifestyle interventions reduce blood cholesterol levels and improve other cardiovascular risk factors, but adherence to these measures is low among the general patient population. Encouraging and motivating patients to *improve adherence to lifestyle measures* may aid in the attainment of treatment goals in cases where the possibility of using lipid-lowering drugs is limited.

The ultimate goal of lipid-lowering treatment is to decrease cardiovascular risk, which depends upon the interplay of multiple risk factors. *Control of other risk factors*—*especially those of hypertension and smoking*—effectively reduces cardiovascular risk, which may move the patient to a lower risk category with less stringent, and more easily attainable, lipid goals.

Coenzyme Q10 (CoQ10) supplementation is frequently used for statin myalgia, but the evidence in support of its use has, thus far, been contradictory. Recently, a well-designed trial [21], in conjunction with a meta-analysis of previous smaller trials [22], consistently failed to demonstrate a difference between CoQ10 and placebo, demonstrating that the *beneficial effect of CoQ10 supplementation in patients with statin*-*induced myalgia is quite unlikely*.

### Statin Therapy

In patients with partial statin intolerance (i.e., those who require, but are unable to tolerate, moderate or high doses of potent statins), lower doses, or less potent statins, should be used.

For patients unable to tolerate any statin at the usual starting daily dose, there is emerging agreement that very low doses, and/or less-than-daily dosing, should be attempted [7, 13]. Since the association between statin dose and low-density cholesterol (LDL-C) is logarithmic, reducing the usual dose of a statin to one-half (or even to one-quarter) still provides a reasonable degree of lipid lowering (ultimately, this approach is simply applying the notorious “rule of six” in the reverse direction). Multiple studies of patients with hypercholesterolemia have demonstrated that rosuvastatin 5–10 mg or atorvastatin 10–20 mg given every other day produced LDL-C reduction of 20–40 % [7]. In patients with previous statin intolerance, rosuvastatin administered once or twice weekly (at a mean dose of 10 mg per week) achieved an LDL-C reduction of 23–29 % and was well tolerated by 74–80 % [23–25] of patients. In a recent review report from a specialized lipid clinic, 90 % of patients referred for intolerance to multiple statins were actually able to tolerate statin therapy, although the majority was at a reduced dose and less-than-daily dosing [26]. Obviously, the efficacy of non-licensed dosing regimens in terms of reducing cardiovascular risk has not been studied. On the other hand, most statin effects are mediated through the lowering of LDL-C; therefore, it would seem reasonable to assume that the cardiovascular risk reduction achieved with alternate statin dosing regimens would be proportionate to their LDL-lowering effects.

In terms of a practical approach to statin intolerance, listening to the patient’s complaints and fears is crucial to encourage greater receptivity and willingness to try various statins and dosing schedules, some of which may be associated with adverse symptoms [5, 26]. These patients need to understand that (i) while these symptoms may be bothersome, they are rarely dangerous, and (ii) they are free to discontinue the drug at any time. It is equally important that physicians emphatically explain the beneficial effects of therapy in terms of cardiovascular event reduction.

In order to prevent unnecessarily intense symptoms of statin intolerance and improve patient adherence to statin therapy in the general population, it may be wise to initiate statin therapy in statin-naive patients with low or moderate (rather than high) doses in the majority of cases, except for patients at highest cardiovascular risk. Patients who previously tolerated lower doses are much more willing to return to them in cases where they experienced problems with higher doses, compared to those who were intolerant to a higher starting dose. It is also advisable to perform both creatine kinase and liver tests prior to commencing statin therapy in order to establish reference baseline values in case the patient develops elevations in these tests during therapy.

### Non-statin Lipid-Lowering Drugs

Other lipid-lowering drugs may be needed to achieve appropriate targets, either in combination with statins, or alone, if statins are not tolerated at all. A combination of these drugs and low-dose statin therapy can provide reductions in LDL-C similar to those obtained with high doses of statins.

Ezetimibe decreases LDL-C by 15–20 % (either in combination with statins or as monotherapy) and is widely used in patients with statin intolerance. Ezetimibe is well tolerated, but the evidence of cardiovascular benefit is limited to one trial that demonstrated a modest 6 % reduction of cardiovascular events [27].

Fibrates are primarily used to lower triglycerides and increase high-density cholesterol; they also decrease LDL-C levels, but to a lesser extent. The effect on LDL-C is more pronounced in patients with hypertriglyceridemia. Accordingly, the reduction of cardiovascular risk with fibrates is only 10 % in unselected patient population, but is substantially greater (≈30 %) in patients with hypertriglyceridemia [28]. As such, fibrates represent a reasonable option in these patients. However, caution must be exercised when combining fibrates with statins, as the combination may increase the risk of myalgia.

Bile acid sequestrants (resins) provide LDL-C reduction that is comparable to that observed with ezetimibe, and they have been proven to reduce cardiovascular events. Resins are safe, but poorly tolerated, due to gastrointestinal side effects. The recently developed colesevelam has fewer side effects and better patient compliance.

Niacin is similar to fibrates relative to its effect on blood lipids, but its use in clinical practice has dropped substantially after two clinical-endpoint trials failed to demonstrate cardiovascular benefits of niacin therapy [29, 30].

PCSK9 inhibitors are a novel class of lipid-lowering drugs; they were approved quite recently in the USA and Europe. They reduce LDL-C levels by ≈ 50 %. Meta-analyses of phase 2 and 3 trials demonstrated a >50 % reduction of cardiovascular events with evolocumab and alirocumab [31, 32], and the results of major clinical trials are eagerly awaited by clinicians. Statin intolerance is one of the approved indications for use of PCSK9 inhibitors.

## Conclusions

Muscle problems and other adverse symptoms associated with statin use are relatively frequent reasons for non-adherence and discontinuation of statin therapy, which can contribute to adverse cardiovascular outcomes. However, most patients who experience objectionable symptoms during statin use are still able to tolerate at least some degree of statin therapy. The clinician’s challenge is therefore to help their patients find their way back to statins.

In essence, this task comprises only a few steps: First, identify patients with unlikely statin intolerance, and who can therefore probably continue with some type of adequate statin therapy. A pragmatic definition of statin intolerance, as outlined above, may be useful in this respect. Second, in cases with statin intolerance, consider very low doses of statins and/or altered dosing regimens. In addition, other lipid-lowering drugs may be needed, in conjunction with changes in lifestyle and better control of other cardiovascular risk factors. With this cautious and multifactorial approach, reasonable improvement in blood lipid levels, as well as a marked reduction in global cardiovascular risk score, can be achieved in most patients.

As simple this approach may appear, it may prove difficult in practice. Listening to patients’ complaints and fears, explaining the benefits of therapy, and motivating patients to try various therapeutic schedules (some of which may be associated with adverse symptoms) are often difficult and time-consuming tasks. From a clinician’s perspective, a successful approach to statin intolerance primarily entails the art of successful communication with the patient.
